# Less fear, better function: the impact of minimally invasive cardiac surgery on kinesiophobia and functional capacity compared to median sternotomy

**DOI:** 10.3389/fmed.2026.1829842

**Published:** 2026-05-14

**Authors:** Zehra Can Karahan, Tayfun Özdem

**Affiliations:** 1 Department of Physiotherapy and Rehabilitation, Faculty of Health Sciences, Atılım University, Ankara, Türkiye; 2 Department of Cardiovascular Surgery, Gülhane Training and Research Hospital, University of Health Sciences, Ankara, Türkiye

**Keywords:** cardiac surgery, functional capacity, kinesiophobia, minimally invasive surgery, sternotomy

## Abstract

**Introduction:**

The comparative impact of Minimally Invasive Cardiac Surgery versus median sternotomy on the interplay between peripheral muscle strength, kinesiophobia, and functional capacity remains unclear. This study compared these parameters in the early postoperative period.

**Methods:**

Forty-six patients (30 Sternotomy, 16 Minimally Invasive Cardiac Surgery) were included in this prospective study. Peripheral muscle strength (handgrip and knee extension), functional capacity (30-Second Sit-to-Stand test), and kinesiophobia (Tampa Kinesiophobia Scale) were assessed preoperatively and on the postoperative 5th day.

**Results:**

Both groups experienced a significant decline in postoperative muscle strength from baseline; however, there was no statistically significant difference between the groups regarding the magnitude of this decline (e.g., right handgrip *p* = 0.366, right knee extension *p* = 0.294). However, the Minimally Invasive Cardiac Surgery group demonstrated superior functional performance in the 30-Second Sit-to-Stand test (*p* = 0.008) and significantly lower increases in kinesiophobia scores compared to the sternotomy group (*p* = 0.008). A significant negative correlation was found between kinesiophobia and functional capacity (*r* = −0.311, *p* = 0.035).

**Conclusion:**

Although Minimally Invasive Cardiac Surgery does not mitigate surgery-associated muscle strength decline, it offers a distinct advantage in preserving functional capacity by minimizing kinesiophobia. Functional limitations after sternotomy appear driven more by movement-related fear than muscle weakness. Consequently, rehabilitation strategies should integrate kinesiophobia management to optimize early mobilization and functional independence.

**Clinical trail registration:**

ClinicalTrials.gov, identifier (NCT07172529).

## 1 Introduction

Cardiac surgery is a cornerstone in the treatment of major cardiovascular conditions such as coronary artery disease and valvular heart disease. While median sternotomy remains the gold standard for accessing the heart, it involves significant surgical trauma, including the division of the sternum, which can lead to postoperative pain, respiratory dysfunction, and prolonged recovery time (1). In recent decades, Minimally Invasive Cardiac Surgery (MICS) has gained increasing popularity. While right mini-thoracotomy is frequently utilized, other approaches such as partial sternotomy or transaxillary incisions may be more dominant depending on the specific type of surgery and institutional protocols. MICS aims to maintain equivalent clinical outcomes while minimizing surgical trauma. Although early literature suggested advantages such as reduced pain and shortened hospital stays compared to conventional sternotomy, these ‘improved’ outcomes remain a subject of intense clinical debate. Furthermore, while studies like Doenst et al. report faster postoperative mobilization associated with MICS, it is heavily debated whether this is a direct result of the surgical technique itself or a consequence of patient selection bias, as healthier patients are often selected for minimally invasive procedures (2, 3).

Despite surgical advancements, the postoperative period is often characterized by a decline in physical function (4). The stress of surgery, systemic inflammation, and postoperative immobilization can lead to significant losses in peripheral muscle mass and strength (5). However, physical recovery is not solely dependent on physiological muscle strength; it is also heavily influenced by psychological factors. Kinesiophobia, defined as an excessive, irrational, and debilitating fear of physical movement and activity resulting from a feeling of vulnerability to painful injury or reinjury, is a prevalent barrier in cardiac patients (6, 7). In current clinical practice, early postoperative mobilization strategies are initiated as soon as patients achieve hemodynamic stability, often utilizing standardized progressive programs even prior to intensive care unit (ICU) discharge. These protocols typically involve a gradual increase in physical activity, progressing from dangling the legs on the edge of the bed to standing and eventual ambulation. Within this early rehabilitation context, the ‘first out-of-bed’ moment represents a highly critical physiological and psychological milestone for both the patient and the attending nurses and physiotherapists (8). However, this crucial step is frequently hindered by movement-related fear. In patients undergoing median sternotomy, the fear of sternal instability or “damaging the bone” often leads to avoidance behaviors, further exacerbating muscle atrophy and delaying functional recovery (9). Although MICS avoids sternal division, thus preserving the structural integrity of the chest wall, it is unclear whether this surgical advantage translates into reduced kinesiophobia and better preservation of functional muscle performance in the early postoperative period (2, 10, 11).

Therefore, the present study aims to compare the effects of minimally invasive cardiac surgery versus median sternotomy on peripheral muscle strength, kinesiophobia levels, and functional capacity. The fifth postoperative day was specifically chosen for assessment as it represents a pivotal functional milestone in acute recovery. By this stage, patients typically transition to the surgical ward and begin independent mobilization. This specific timeframe provides an optimal window to evaluate how movement-related fear influences functional independence prior to hospital discharge, while also aligning with clinical benchmarks for early postoperative muscle wasting. By revealing how different surgical approaches affect the patient’s psychophysical status, this study seeks to provide evidence for tailoring more effective, surgery-specific rehabilitation strategies.

## 2 Materials and methods

### 2.1 Study design and participants

This prospective observational study was conducted at Gülhane Education and Research Hospital Cardiovascular Surgery Clinic between June 2024 and February 2025. Patients aged between 43 and 79 years who underwent elective cardiac surgery, including coronary artery bypass grafting (CABG) and/or valve surgery, were recruited. Inclusion criteria were the ability to ambulate independently prior to surgery, absence of neuromuscular or orthopedic disorders, and cognitive competence to follow verbal commands. Patients with revision surgeries, extended ICU stays (>2 days), or diagnosed neurological or psychiatric conditions and deep venous thrombosis were excluded.

### 2.2 Clinical and demographic data collection

In addition to the primary outcome measures, detailed demographic and clinical characteristics were recorded for each participant. The surgical details encompassed the following metrics: the type of incision (median sternotomy or minimally invasive approach), aortic cross-clamp time, total cardiopulmonary bypass duration, total operative time, length of stay in the intensive care unit, and total hospital stay. Preoperative assessments encompassed the presence of chronic comorbidities, age, height, weight, body mass index (BMI), and cardiovascular risk factors. The perioperative symptoms, including dyspnea, cough, sputum production, and fatigue, were evaluated using a dichotomous scale (present/absent) both before and after surgery. Postoperative analgesia was provided through a standardized multimodal regimen to ensure consistency between surgical groups. Pain severity was assessed using a 10-cm Visual Analog Scale (VAS) during both rest and activity. Analgesic administration was titrated to maintain VAS scores below 4, particularly prior to functional assessments and mobilization, thereby minimizing the impact of acute pain on kinesiophobia and physical performance.

### 2.3 Ethical approval

The study protocol was approved by Scientific Research and Publication Ethics Committee of Çankırı Karatekin University (Approval Number: 3dcd45b218264a9a, Date: 11.05.2024). Written informed consent was obtained from all participants, in accordance with the Declaration of Helsinki. The study was registered at ClinicalTrials.gov (NCT07172529).

### 2.4 Outcome measures

#### 2.4.1 Peripheral muscle strength

Quadriceps muscle strength of both lower limbs was assessed using the K-Force Muscle Tester (Kinvent, France), a hand-held digital dynamometer. Standardized sitting position was used, and the participants performed three maximal isometric contractions with a 30-s rest between each trial; the highest value was recorded (12).

Grip strength was measured for both hands using a Lafayette Hydraulic Hand Dynamometer (Model 78,010). Participants were seated with the shoulder adducted and neutrally rotated, elbow flexed at 90°, forearm in neutral position, and wrist between 0–30° dorsiflexion. The average of three attempts was used for analysis.

#### 2.4.2 Functional capacity

Functional capacity was evaluated using the 30-Second Sit-to-Stand Test (30s-SST). Participants were instructed to stand up and sit down from a standard chair as many times as possible in 30 s without using their arms (13). The number of correctly completed repetitions was recorded. In patients who have had a heart attack, the cutoff value for this test has been found to be 13 repetitions (14).

#### 2.4.3 Kinesiophobia

Kinesiophobia was assessed using the Turkish version of the 17-item Tampa Scale for Kinesiophobia (TSK-17), a self-reported questionnaire evaluating fear of movement or re-injury. Items are rated on a 4-point Likert scale (1–4), yielding total scores between 17 and 68, with higher scores indicating greater kinesiophobia (15, 16).

### 2.5 Statistical analysis

Data analysis was performed using IBM SPSS Statistics 26.0 (IBM Corp., Armonk, NY, USA). Continuous variables were presented as mean ± standard deviation, and categorical variables as frequencies and percentages. The Shapiro–Wilk test indicated non-normal distribution; therefore, non-parametric tests were applied. The Mann–Whitney U test was used for between-group comparisons of continuous variables and difference scores, with effect sizes calculated as eta-squared. Fisher’s Exact Test was used to compare categorical demographic data. Intra-group preoperative to postoperative changes in symptoms were analyzed using the McNemar test. Relationships between variables were assessed using Spearman’s rank correlation coefficient. A *p*-value <0.05 was considered statistically significant. To evaluate the statistical robustness of the findings, a post-hoc power analysis was performed using G*Power software (version 3.1.9.7). Based on the included sample sizes (*n* = 30 for the Sternotomy group and n = 16 for the MICS group) and the observed large effect size (η^2^ = 0.13; Cohen’s *d* = 0.77) for the primary outcomes of kinesiophobia and functional capacity, the achieved power (1-*β*) was calculated as 68.5% at a significance level of *α* = 0.05. Although it is acknowledged that the study is underpowered by standard criteria, it successfully detected the observed large effect sizes (η^2^ = 0.13). Consequently, findings regarding non-significant parameters must be interpreted with caution due to the inherent risk of Type II errors associated with the restricted sample size in the MICS group. Additionally, an ANCOVA was performed to control baseline BMI differences as a potential confounder.

## 3 Results

Initially, a total of 49 patients were recruited for the study. However, one patient was excluded due to surgery cancellation, and two patients were excluded from the final analysis due to postoperative mortality. Consequently, a total of 46 patients (30 in the Sternotomy group and 16 in the MICS group) completed the study and were included in the statistical analysis. Demographic and baseline clinical characteristics of the study population are summarized in Table 1.

**Table 1:** Demographic and clinical characteristics of patients.

Variables		Sternotomy (*n* = 30)	MICS (*n* = 16)	*p*
		*X* ± SD	*X* ± SD	
Age (year)		60.33 ± 8.23	62.50 ± 8.36	0.196^a^
BMI		27.57 ± 3.74	31.70 ± 5.45	0.009^a^
		*n* (%)	*n* (%)	
Gender	Female	5 (16.7%)	7 (43.8%)	0.770^b^
	Male	25 (83.3%)	9 (56.3%)	
Marital Status	Married	30 (100%)	13 (81.3%)	**0.037**^**b**^
	Single	0	3 (18.8%)	
Educational Level	Illiterate	1 (3.3%)	1 (6.3%)	0.554^b^
	Primary School	0	1 (6.3%)	
	Middle School	15 (50%)	7 (43.8%)	
	High School	8 (26.7%)	6 (37.5%)	
	Undergraduate	5 (16.7%)	1 (6.3%)	
	Postgraduate	1 (3.3%)	0	
Occupation	Unemployed	0	1 (6.3%)	0.253^b^
	Homemaker	3 (10.0%)	5 (31.3%)	
	Blue-collar Worker	1 (3.3%)	1 (6.3%)	
	Civil Servant	3 (10.0%)	1 (6.3%)	
	Retired	17 (56.7%)	7 (43.8%)	
	Private Sector Employee	6 (20.0%)	1 (6.3%)	
Chronic disease	Yes	22 (73.3%)	13 (81.3%)	0.722^b^
	No	8 (26.7%)	3 (18.8%)	
Family History	Yes	13 (43.3%)	3 (18.8%)	0.117^b^
	No	17 (56.7%)	13 (81.3%)	
Smoking	Yes	14 (46.7%)	8 (50.0%)	1^b^
	No	16 (53.3%)	8 (50.0%)	
Alcohol Consumption	Yes	4 (13.3%)	1 (6.3%)	0.645^b^
	No	26 (86.7%)	15 (95.8%)	
Hypertension	Yes	16 (53.3%)	12 (75.0%)	0.210^b^
	No	14 (46.7%)	4 (25.0%)	
Diabetes Mellitus	Yes	11 (36.7%)	5 (31.3%)	0.757^b^
	No	19 (63.3%)	11 (68.8%)	
Dyslipidemia	Yes	3 (10.0%)	4 (25.0%)	0.216^b^
	No	27 (90.0%)	12 (75.0%)	
Obesity	Yes	14 (46.7%)	10 (62.5%)	0.364^b^
	No	16 (53.3%)	6 (37.5%)	
Type of surgery	CABG	23 (76.7%)	13 (81.3%)	0.904^b^
	MVR	3 (10.0%)	1 (6.3%)	
	AVR	4 (13.3%)	2 (12.5%)	

Values are expressed as mean ± standard deviation or number (percentage). MICS, Minimally Invasive Cardiac Surgery; BMI, Body Mass Index. CABG, Coronary artery bypass graft, MVR, Mitral Valve Replacement, AVR, Atrial Valve Replacement. ^a^Mann–Whitney U Test; ^b^Fisher’s Exact Test (*χ*^2^).

The two groups were comparable in terms of mean age, gender distribution, education level, and occupation. Similarly, no significant differences were observed regarding preoperative comorbidities (hypertension, diabetes, dyslipidemia) or habits (smoking, alcohol consumption). However, significant differences were found in BMI and marital status. The MICS group had a significantly higher mean BMI compared to the Sternotomy group (31.70 ± 5.45 vs. 27.57 ± 3.74 kg/m^2^; *p* = 0.009). Regarding marital status, all patients in the Sternotomy group were married, whereas 18.8% of the MICS group were single (*p* = 0.037).

Table 2 details the changes in clinical symptoms from the preoperative to the postoperative period. In both the Sternotomy and MICS groups, a statistically significant increase was observed in the prevalence of cough and sputum production following surgery. Specifically, cough complaints rose from 20.0 to 76.7% in the Sternotomy group (*p* < 0.001) and from 12.5 to 68.8% in the MICS group (*p* = 0.012). Similarly, sputum production increased significantly in both groups (Sternotomy *p* < 0.001; MICS *p* = 0.003). Conversely, there were no statistically significant changes in dyspnea (Sternotomy *p* = 0.180; MICS *p* = 1.000) or fatigue levels (Sternotomy *p* = 0.267; MICS *p* = 0.125) in either group when comparing preoperative and postoperative assessments.

**Table 2:** Comparison of preoperative and postoperative symptoms within groups.

Symptoms	Group	Preoperative		Postoperative		*p*
		Present *n* (%)	Absent *n* (%)	Present *n* (%)	Absent *n* (%)	
*Dyspnea*	Sternotomy	11 (36.7%)	19 (63.3%)	6 (20.0%)	24 (80.0%)	0.180
	MICS	4 (25.0%)	12 (75.0%)	4 (25.0%)	12 (75.0%)	1
*Cough*	Sternotomy	6 (20.0%)	24 (80.0%)	23 (76.7%)	7 (23.3%)	**<0.001**
	MICS	2 (12.5%)	14 (87.5%)	11 (68.8%)	5 (31.3%)	**0.012**
*Sputum*	Sternotomy	8 (26.7%)	22 (73.3%)	26 (86.7%)	4 (13.3%)	**<0.001**
	MICS	2 (12.5%)	14 (87.5%)	13 (81.3%)	3 (18.8%)	**0.003**
Fatigue	Sternotomy	14 (46.7%)	16 (53.3%)	19 (63.3%)	11 (36.7%)	0.267
	MICS	4 (25.0%)	12 (75.0%)	8 (50.0%)	8 (50.0%)	0.125

MICS, Minimally Invasive Cardiac Surgery. McNemar test. Bold values indicate statistical significance at the *p* < 0.05 level.

The intraoperative timelines and postoperative recovery periods for both surgical approaches are detailed in Table 3. There were no statistically significant differences between the Sternotomy and MICS groups regarding intraoperative parameters, including cardiopulmonary bypass (CPB) time, cross-clamp time, total operation time, and anesthesia duration. Similarly, postoperative recovery times were comparable; the length of stay in the ICU and the total length of hospital stay did not differ significantly between the surgical approaches (*p* = 0.206 and *p* = 0.144, respectively).

**Table 3:** Comparison of operative and postoperative durations between groups.

Variables	Sternotomy (*n* = 30)	MICS (*n* = 16)	*Z*	*p*
	*X* ±SD	X ±SD		
ICU stay (days)	1.25 ± 0.51	1.46 ± 0.63	−1.265	0.206
Length of hospital stay (days)	6.78 ± 1.59	6.53 ± 2.19	−1.459	0.144
CPB time (min)	124.78 ± 47.72	150.53 ± 73.08	−0.727	0.467
Cross-clamp time (min)	79.21 ± 31.16	85.80 ± 32.75	−0.346	0.729
Operation time (min)	271.60 ± 67.77	266.00 ± 61.91	−0.297	0.766
Anesthesia time (min)	282.67 ± 67.81	279.73 ± 66.58	−0.051	0.959

Values are expressed as mean ± standard deviation. MICS, Minimally Invasive Cardiac Surgery; ICU, Intensive Care Unit; CPB, Cardiopulmonary bypass (total perfusion time). Mann–Whitney *U*-test.

Table 4 presents the comparison of the change scores (ΔPostoperative - ΔPreoperative) between the two surgical approaches. There were no statistically significant differences between the Sternotomy and MICS groups regarding the changes in pain levels, handgrip strength (right/left), or knee extension strength (right/left). This indicates that the physiological impact of surgery on isometric muscle strength was similar in both groups.

**Table 4:** Comparison of the change scores (postoperative–preoperative) between groups.

Variables		Between- group difference		
	Mean difference (95% CI)	*Z*	*p*-value	Effect size (eta-square)
Pain	−2.49 (−3.22−1.76)	−1.008	0.314	
30-s STS test	−2.23 (−2.91−1.56)	−2.643	**0.008**	**0.13**
Right handgrip strength	−4.58 (−7.06−2.11)	−0.903	0.366	
Left handgrip strength	−3.59 (−5.18−2.00)	−0.162	0.871	
Right knee extension strength	−1.15 (−1.75−0.54)	−1.050	0.294	
Left knee extension strength	−0.78 (−1.28−0.28)	−1.213	0.225	
Kinesiophobia	2.52 (1.22–3.82)	−2.651	**0.008**	**0.13**

Values represent the difference in change scores between the Sternotomy and MICS groups. CI, Confidence Interval; STS, Sit-to-Stand. *p*-values were obtained using the Mann–Whitney U test. Effect size calculated as eta-squared. To account for baseline BMI discrepancies, an ANCOVA was performed. After adjusting for BMI, the main effect of incision type remained significant for both the 30-s STS test (*F* = 4.391, Adjusted *p* = 0.042) and Kinesiophobia (*F* = 9.049, Adjusted *p* = 0.004). Bold values indicate statistical significance at the *p* < 0.05 level.

However, significant differences were observed in functional capacity and kinesiophobia. The MICS group demonstrated significantly better preservation of functional performance in the 30-s STS test compared to the Sternotomy group (*p* = 0.008). Furthermore, the increase in kinesiophobia scores was significantly lower in the MICS group (*p* = 0.008). For both significant variables, the effect size was calculated as 0.13. To address the potential confounding effect of baseline BMI discrepancies, an ANCOVA analysis was performed with BMI included as a covariate. After adjusting for BMI, the main effect of incision type remained statistically significant for both the 30-s STS test (*F*(1, 43) = 4.391, *p* = 0.042, η^2^ = 0.093) and kinesiophobia (*F*(1, 43) = 9.049, *p* = 0.004, η^2^ = 0.174), indicating that the observed functional and psychological advantages of MICS persist independent of body mass.

Table 5 shows the correlation analysis between the study variables. A significant relationship was found between the type of surgery and psychophysical outcomes. The incision type showed a moderate positive correlation with the 30-s STS test (*r* = 0.394, *p* = 0.007) and a moderate negative correlation with kinesiophobia (*r* = −0.395, *p* = 0.007). These findings indicate that the minimally invasive approach is associated with higher functional performance and lower levels of kinesiophobia.

**Table 5:** Spearman’s rank correlation analysis between variables.

Variables	Pain *r* (*p*)	30-s STS (*p*)	Right HGS *r* (*p*)	Left HGS *r* (*p*)	Right KES *r* (*p*)	Left KES *r* (*p*)	Kinesiophobia *r* (*p*)
Incision type	−0.150 (0.319)	0.394** (0.007)	−0.135 (0.372)	0.024 (0.873)	−0.157 (0.299)	−0.181 (0.229)	−0.395** (0.007)
Pain		0.136 (0.369)	0.155 (0.303)	0.108 (0.473)	0.227 (0.129)	0.274 (0.066)	−0.123 (0.414)
30-s STS			−0.009 (0.954)	0.134 (0.376)	0.073 (0.631)	−0.006 (0.967)	−0.311* (0.035)
Right HGS				0.633** (<0.001)	0.352* (0.016)	0.286 (0.054)	0.179 (0.233)
Left HGS					0.378** (0.010)	0.206 (0.170)	0.134 (0.376)
Right KES						0.709** (<0.001)	0.185 (0.219)
Left KES							0.291 (0.050)

STS, Sit-to-Stand Test; HGS, Handgrip Strength; KES, Knee Extension Strength. **p < 0.01, *p < 0.05. Bold values indicate statistical significance at the *p* < 0.05 level.

Crucially, a significant negative correlation was observed between kinesiophobia and the 30-s STS test (*r* = −0.311, *p* = 0.035), suggesting that higher fear of movement is associated with reduced functional capacity.

Regarding muscle strength, significant positive correlations were found between upper and lower extremity strengths (e.g., right handgrip and right knee extension, *p* < 0.001), confirming the internal consistency of muscle measurements. However, no significant correlation was found between muscle strength and kinesiophobia (e.g., right handgrip *p* = 0.233, right knee extension *p* = 0.219), indicating that kinesiophobia limits functional mobility (STS) rather than physiological muscle force generation.

## 4 Discussion

Our study is among the first to investigate the interplay between peripheral muscle strength, kinesiophobia, and functional capacity in patients undergoing minimally invasive cardiac surgery (MICS) compared to conventional median sternotomy. The most significant finding is that, although both surgical approaches resulted in comparable postoperative peripheral muscle strength (handgrip and knee extension), patients in the MICS group demonstrated significantly lower levels of kinesiophobia and superior functional capacity. These results suggest that the primary early-recovery advantage of MICS may not stem from preserving physiological muscle force, but rather from reducing movement-related fear, thereby allowing patients to utilize their existing muscle capacity more effectively.

### 4.1 Peripheral muscle strength

It is well documented that patients undergoing cardiac surgery experience a decline in peripheral muscle strength due to systemic inflammation, surgical stress, and postoperative inactivity (17–19). Consistent with this, our study found no significant difference in handgrip or knee extension strength reductions between the MICS and sternotomy groups. This decline aligns with previous literature highlighting the catabolic impact of cardiac surgery. For instance, Dimopoulos et al. demonstrated a trend toward decreased skeletal quadriceps muscle thickness following intensive care unit (ICU) admission post-cardiac surgery (19). They further reported that this muscle wasting was significantly associated with prolonged mechanical ventilation and extended ICU stays. In the present study, the comparable decline in quadriceps strength across both groups supports the hypothesis that postoperative muscle weakness is driven primarily by systemic factors and immobilization during the critical care period, rather than the specific surgical incision technique.

### 4.2 Functional capacity

Despite comparable declines in isometric muscle strength, a striking disparity in functional capacity was observed between the groups. The 30-s STS test, which mimics the essential daily activity of rising from a chair, revealed significantly better performance in the MICS group compared to the sternotomy group. This finding is consistent with a study by Mosceralli et al., who reported that patients undergoing minimally invasive valve surgery achieved faster physical recovery and returned to daily activities sooner than those undergoing conventional sternotomy (20). The superior STS performance in our MICS cohort—despite similar quadriceps strength—suggests that these patients were better able to translate their physiological capacity into functional movement. This highlights that early clinical recovery depends not solely on muscle force, but also on the ability to mobilize without mechanical or psychological inhibition. Although BMI differed significantly between the groups at baseline, our additional multivariate analyses controlling for BMI indicated that the observed functional disparities remained significant, suggesting that BMI did not account for these superior clinical findings in the MICS cohort.

The observed disparity in functional capacity, despite similar muscle strength declines, suggests that mechanical and psychological factors play a more dominant role in early recovery than physiological force alone. In patients undergoing median sternotomy, the fear of sternal dehiscence often leads to a restrictive ‘fear-avoidance’ cycle, which significantly limits functional independence. Our findings are strongly reinforced by the recent work of Mehani et al. (2024), who demonstrated that incorporating graduated, bilateral, and unilateral upper limb exercises combined with integrated abdominal contractions is both safe and effective for patients’ post-CABG (21). By utilizing such targeted stabilization exercises to approximate sternal edges and reduce separation, clinicians can directly mitigate postoperative pain and enhance performance in activities of daily living. Integrating these evidence-based stabilization techniques into early inpatient rehabilitation may provide the structural security necessary to break the cycle of kinesiophobia, thereby optimizing the transition from clinical stability to functional independence in the acute postoperative phase.

### 4.3 Kinesiophobia

Crucially, while physiological muscle decline was comparable, the psychological response to surgery differed significantly between the groups. Our results indicated that patients in the MICS group exhibited significantly lower kinesiophobia scores than those undergoing median sternotomy. Sternotomy involves the complete division of the sternum, which often instills a specific fear of sternal instability, clicking sensations, or wound dehiscence during mobility tasks. This fear creates a potent psychological barrier that drives fear-avoidance behavior, leading patients to limit their physical activity to protect the surgical site. In contrast, preserving the structural integrity of the thoracic cage in MICS likely provides patients with a greater sense of physical security. This reduction in movement-related fear explains why MICS patients achieved significantly better results in the 30-s STS test, effectively translating their available muscle strength into functional movement.

Interestingly, regarding the relationship between postoperative pain severity and kinesiophobia scores, no significant correlation was detected, suggesting kinesiophobia may represent a distinct psychological barrier likely rooted in concerns over mechanical sternal stability and the perceived vulnerability of the thoracic cage. This finding further emphasizes that managing postoperative recovery requires more than just effective analgesia; it necessitates targeted cognitive-behavioral strategies and structural reassurance to break the fear-avoidance cycle.

An interesting demographic observation in our cohort was the significant BMI in the MICS group. Generally, a higher body mass imposes a biomechanical disadvantage, often correlating with poorer performance in weight-bearing functional assessments such as the 30-s STS test (22). Strikingly, despite this baseline physical disadvantage, the MICS group still achieved superior functional mobility compared to the sternotomy group. This observation further strengthens our premise: the reduction in movement-related fear and the preservation of thoracic stability associated with MICS exert such a profound positive effect on early recovery that they overcome the inherent functional burdens of a higher BMI.

### 4.4 Systemic respiratory burdens and functional mobility

It is also worth noting that, regardless of the surgical incision size, patients in both groups experienced a significant increase in respiratory symptoms—specifically cough and sputum production—in the early postoperative period. This finding aligns with established literature indicating that general anesthesia, cardiopulmonary bypass, and mechanical ventilation induce temporary mucociliary dysfunction and airway irritation (23, 24). The persistence of these respiratory challenges in the MICS group suggests that while the mini-thoracotomy approach preserves chest wall stability and reduces kinesiophobia, it does not completely eliminate the systemic respiratory side effects of cardiac surgery. However, despite these shared respiratory burdens, the MICS group still demonstrated superior functional mobility, further emphasizing the dominant role of incisional pain and fear in limiting early recovery following sternotomy.

Although our results underline the distinct early postoperative advantages of MICS in mitigating kinesiophobia and maintaining functional mobility, it is essential to consider that the fifth postoperative day provides only a snapshot of the acute recovery phase. How these psychological and functional disparities evolve throughout the broader recovery trajectory remains to be fully elucidated. Consequently, we advocate for future longitudinal studies designed to track kinesiophobia and functional recovery over the mid- to long-term (e.g., at 3, 6, and 12 months post-surgery). Such extended follow-up is critical to determining whether the early functional gains observed in the MICS group translate into a sustained clinical lead, or whether median sternotomy patients eventually achieve functional parity once sternal stability is established and fear-avoidance behaviors naturally diminish.

In conclusion, the findings of this study suggest that while cardiac surgery inevitably leads to a decline in peripheral muscle strength regardless of the surgical technique, MICS offers a distinct advantage in preserving functional capacity during the early postoperative period. This functional superiority appears to be driven not by greater muscle strength, but by significantly lower levels of kinesiophobia. These results have important clinical implications for postoperative rehabilitation; they indicate that early physical recovery is often limited by psychological barriers rather than physiological muscle weakness alone. Therefore, rehabilitation protocols—especially for patients undergoing median sternotomy—should integrate strategies to identify and manage movement-related fear to optimize early mobilization and functional independence.

## 5 Limitations

This study has several limitations that should be acknowledged. First, the single-center design and relatively small sample size in the MICS group (n = 16) may limit the generalizability of our findings. While our post-hoc analysis indicated sufficient power for primary outcomes, a risk of Type II errors remains for secondary parameters, such as peripheral muscle strength. Second, the MICS group had a significantly higher baseline BMI. Although an ANCOVA was utilized to adjust for BMI and confirm our primary findings, the non-normal distribution of our data and the relatively small sample size should be considered when interpreting these adjusted results; future large-cohort studies are warranted to further validate these models. Third, although patients were managed with standardized analgesia, movement-related fear is inherently intertwined with postoperative pain; the exact interplay between acute incisional pain and kinesiophobia remains a complex variable. Finally, our follow-up was restricted to the early postoperative phase (day 5). Future multi-center, longitudinal studies are needed to determine whether the observed differences in kinesiophobia and functional recovery persist in the mid- to long-term stages.

## 6 Conclusion

This study demonstrates that while MICS and conventional median sternotomy result in comparable early postoperative declines in peripheral muscle strength, the MICS approach offers superior preservation of functional capacity. Our findings indicate that this functional advantage is primarily driven by significantly lower levels of kinesiophobia rather than the preservation of physiological muscle strength. Consequently, although MICS does not prevent the catabolic effects of surgery on muscle force, it facilitates early mobilization by reducing movement-related fear. These results highlight the critical need to integrate psychosocial evaluations and kinesiophobia management into postoperative rehabilitation protocols—particularly for patients undergoing median sternotomy—to optimize functional recovery and independence. While the present findings demonstrate a substantial early-phase benefit for the MICS approach, further longitudinal studies are necessary to ascertain the long-term stability of reduced kinesiophobia levels. Tracking these trajectories beyond the acute hospital stay will provide a more comprehensive understanding of how early psychological interventions and surgical choices collectively influence long-term functional independence and quality of life in cardiac patients.

## Data Availability

The raw data supporting the conclusions of this article will be made available by the authors, without undue reservation.
